# Novel and Emerging Therapies for Inflammatory Bowel Disease

**DOI:** 10.3389/fphar.2021.651415

**Published:** 2021-04-14

**Authors:** Badr Al-Bawardy, Raina Shivashankar, Deborah D. Proctor

**Affiliations:** ^1^ Section of Digestive Diseases, Yale School of Medicine, New Haven, CT, United States; ^2^ Division of Gastroenterology and Hepatology, Thomas Jefferson University, Philadelphia, PA, United States

**Keywords:** inflammatory bowel disease, small molecule, biologic, novel therapy, emerging therapy

## Abstract

Inflammatory bowel diseases (IBD) such as ulcerative colitis and Crohn’s disease are chronic, relapsing and remitting disorders of intestinal inflammation with potential systemic manifestations. Despite the availability of current biologics, such as anti-tumor necrosis factor (anti-TNF), anti-integrins, anti-interleukins and small molecules such as tofacitinib, the rates of primary and secondary treatment failure remain high in IBD. This highlights the importance of continued development of new therapeutic targets and modifications of existing ones to improve the treatment response rates and to also improve the safety profile and tolerability of these medications. In this review we will discuss novel treatment target agents including selective janus kinase (JAK) inhibitors, anti-interleukin (IL) (IL-12/IL-23), leukocyte trafficking/migrating inhibitors (such as sphingosine-1-phosphate receptor modulator) and other small molecules currently in development.

## Introduction

Inflammatory bowel disease (IBD) is a chronic, progressive disease that can lead to complications including bowel damage, need for hospitalizations and surgery, decreased quality of life, and disability. The incidence rates of IBD have been increasing world-wide. While the highest rates have been noted in North America and Europe, increasing incidence rates have been observed in previously low-incidence regions such as Asia as these countries become more developed (Loftus Jr, 2004; Molodecky et al., 2012; Shivashankar and Lewis, 2017a). The cause for this phenomenon is largely unknown, however it may be related to the complex interplay between genetics and the environment (Shivashankar and Lewis, 2017a). The latter has been implicated in the development of IBD because of the observation that higher incidence rates are seen in industrialized nations; therefore diet, pollution, microbial exposure, and sanitation may be involved (Molodecky and Kaplan, 2010; Shivashankar and Lewis, 2017a).

Since IBD can be a chronic and progressive disease, optimal treatment early in the disease course to prevent complications is paramount. The current treatment paradigm for moderate-severe Crohn’s disease (CD) and ulcerative colitis (UC) is to use biologic therapy early to achieve clinical remission and mucosal healing, which will ultimately decrease the risk of corticosteroid use, surgeries, hospitalizations and increase quality of life (Klenske et al., 2019).

The currently available biologic agents include anti-tumor necrosis factor-alpha (TNF-α) agents (infliximab, adalimumab, certolizumab, and golimumab); anti-integrin agents (vedolizumab, natalizumab); and an anti-interleukin (IL) 12-23 agent (ustekinumab). Finally, tofacitinib, a non-biologic small molecule is approved for the treatment of moderate-severe UC. The choice between these agents depends on IBD phenotype and behavior, previous biologic exposure and response, potential adverse effects of therapy, patient co-morbidities, and shared-decision making with patients.

Despite the number of available medications, there are appreciable rates of primary non-response, loss of response, or adverse reactions thereby necessitating additional treatment options. Therefore, the purpose of this review article is to describe novel and emerging therapies for IBD. In this review we will discuss novel treatment target agents including selective janus kinase (JAK) inhibitors, anti-interleukin (IL) (IL-12/IL-23), leukocyte trafficking/migrating inhibitors (such as sphingosine-1-phosphate receptor modulator) and other small molecules currently in development (Table 1). This review will focus on the pipeline of these new therapeutic agents and will not include other novel and emerging therapeutic modalities such as stem cell therapy or microbiome targeted therapies.

**TABLE 1:** Target, mode of delivery and development phase of emerging therapeutic agents in IBD.

Drug class	Agent	Target	Mode of delivery	Crohn’s disease	Ulcerative colitis
JAK inhibitors	Tofacitinib	JAK1/JAK3	Oral	N/A	FDA approved
	Filgotinib	JAK1	Oral	Phase III recruiting	Phase IIb/III completed
	Upadacitinib	JAK1	Oral	Phase III recruiting	Phase III recruiting
	TD-1473	Pan-JAK (gut selective)	Oral	Phase II recruiting	Phase IIb/III recruiting
	Brepocitinib (PF-06700841)	TYK2/JAK1	Oral	Phase IIa recruiting	Phase IIb recruiting
	PF-06651600	JAK3	Oral		
	BMS-986165	TYK2	Oral	Phase II recruiting	Phase II recruiting
Anti-trafficking therapies	Vedolizumab SC	α4β7 integrin	SC	N/A	Phase III completed
	Etrolizumab	α4β7 and αEβ7 integrins	SC	Phase III recruiting	Phase III completed
	AJM300	α4 integrin	Oral	N/A	Phase III recruiting
	PF-00547659	MAdCAM	SC	Phase II completed	Phase II completed
IL-23 inhibitors	Risankizumab	IL23/p19 subunit	IV, SC	Phase III active, not recruiting	Phase III enrolling by invitation
	Brazikumab	IL23/p19 subunit	IV, SC	Phase IIb/III recruiting	Phase 2/OLE enrolling by invitation
	Mirikizumab	IL23/p19 subunit	IV, SC	Phase III recruiting	Phase III recruiting
	Guselkumab	IL23/p19 subunit	IV, SC	Phase II/III recruiting	Phase II/III recruiting
S1P receptor modulators	Ozanimod	S1PR1 and S1PR5	Oral	Phase III recruiting	Phase III completed
	Etrasimod	S1PR1, S1PR4 and S1PR5	Oral	Phase II/III recruiting	Phase III recruiting
	Amiselimod (MT-1303)	S1PR1	Oral	Phase II completed	N/A
PDE4 inhibitors	Apremilast	PDE4	Oral	N/A	Phase II completed
TLR9 agonist	Cobitolimod	TLR9	Topical (enema)	N/A	Phase IIb completed, Phase III planned

JAK, janus kinase; TYK 2, tyrosine kinase 2; S1P, sphingosine 1 phosphate; S1PR sphingosine 1 phosphate receptor; PDE4, phosphodiesterase 4; TLR9, toll-like receptor 9; α4β7, alpha4-beta7; αEβ7, alphaE-beta7; α4, alpha4; MAdCAM, mucosal addressin cell adhesion molecule-1; IL-23, interleukin 23; IV, intravenous; SC, subcutaneous; OLE, open label extension.

### JAK Inhibitors

Cytokine mediators of inflammation in IBD such as IL-9, IL-12, IL-23 and interferon-gamma (IFN-γ) are reliant on the Janus Kinase Signal Transducer and Activator of Transcription (JAK-STAT) pathway signaling (Salas et al., 2020). In addition, genome wide association studies have demonstrated risk loci in the JAK region in IBD (Barrett et al., 2008; Anderson et al., 2011). Therefore, targeting the JAK-STAT is an appealing therapeutic modality in IBD. JAK1, JAK2, JAK3 and tyrosine kinase 2 (TYK2) are all part of the JAK family of tyrosine kinase proteins.

#### Tofacitinib

Tofacitinib, a non-selective JAK inhibitor, was approved by the US food and drug administration (FDA) in May 2018 for the treatment of UC. The pivotal induction and maintenance clinical trials, OCTAVE 1-3, have demonstrated efficacy of tofacitinib in inducing and maintaining clinical and endoscopic remission in UC (Sandborn et al., 2017b). Notably, tofacitinib was also shown to be effective in the more refractory anti-TNF-α exposed UC patients compared to placebo. On the other hand, a phase IIb randomized clinical trial of tofacitinib in CD failed to meet the primary endpoints (Panés et al., 2017). Multiple factors might have contributed to the negative results of the study including: study design and high placebo response rate. Subsequently, a systematic review and meta-analysis of clinical trials of different JAK inhibitors (tofacitnib, filgotinib, pefecitinib, upadacitinib, TD-1473) in CD demonstrated the effectiveness of JAK inhibitors in inducing clinical remission (Ma et al., 2019).

Tofacitinib provided the advantage being an oral agent compared to current intravenous (IV) or subcutaneous (SC) biologic agent formulations. The rapid onset of action of tofacitinib is a positive feature as improvement in symptoms of rectal bleeding and stool frequency can occur within 3 days of starting treatment in UC patients according to post-hoc analysis of the pivotal trials (Hanauer et al., 2019). Conversely, tofacitinib has been associated with increased risk of herpes zoster infection and hyperlipidemia (Winthrop et al., 2018). In addition, tofacitinib has a black box warning for venous thromboembolism and mortality after risk was noted in a subset of patients with rheumatoid arthritis taking the 10 mg p.o. BID dose (FDA Drug Safety Communication, 2019). Therefore, there has been an increased interest in the development of more selective JAK inhibitors to enhance the effectiveness and the safety profile in IBD.

#### Filgotinib

Filgotinib is a once daily oral small molecule with higher selectivity for JAK 1 inhibition. JAK 1 (in addition to JAK 3) have been shown to be expressed on both B cells and T cells within the healthy colon mucosa (Salas et al., 2020). The efficacy of filgotinib in CD was evaluated in a phase II, double-blinded, placebo controlled randomized clinical trial, FITZROY. In this trial, patients were randomized to filgotinib 200 mg once a day or placebo and after 10 weeks based on response, patients received either once daily filgotinib 200 mg, filgotinib 100 mg or placebo for another 10 weeks (Vermeire et al., 2017b). A significantly higher proportion of patients in the filgotinib groups achieved the primary endpoint of clinical remission [defined as Crohn’s disease activity index (CDAI) < 150] at week 10 compared to placebo (47 vs. 23%; *p* = 0.0077) (Vermeire et al., 2017b). In anti-TNF-α naïve patients, the rate of clinical remission at week 10 was 60% (n = 34) compared to 37% (n = 26) in anti-TNF-α experienced patients. The rate of endoscopic response and mucosal healing in the overall filgotinib group was numerically higher but did not reach statistical significance. However, endoscopic assessment in this trial was limited by short follow up time. In terms of safety, up to 20 weeks follow up, serious infections were noted in 3% in the filgotinib group with none reported in the placebo group.

A phase IIB/III study (SELECTION) evaluating filgotinib for induction and maintenance therapy for moderate to severe UC was recently completed (Feagan et al., 2020b). The induction study included a cohort of biologic exposed and another cohort of biologic naïve patients (Feagan et al., 2020b; Peyrin-Biroulet et al., 2020). In each cohort patients were randomized to either filgotinib 100 mg, filgotinib 200 mg or placebo daily. The composite primary endpoint of endoscopic, rectal bleeding and stool frequency (EBS) remission (defined as Mayo endoscopic subscore ≤1, rectal bleeding subscore of 0, stool frequency subscore decrease of 1 or more points from baseline and ≤1) rates were significantly higher in the 200 mg filgotinib groups (26.1% in the biologic naïve cohort and 11.5% in the biologic experienced cohort) compared to the placebo groups. One case of pulmonary embolism in the filgotinib 200 mg group was noted and four total cases of herpes zoster in filgotinib groups were noted (1 in the 100 mg group and three in the 200 mg group) (Feagan et al., 2020b).

Patients who achieved clinical remission or response after 10 weeks of induction therapy with either filgotinib or placebo were included in the maintenance study (Peyrin-Biroulet et al., 2020). In this study 664 patients with moderate to severe UC who received placebo induction remained in the placebo maintenance arm, while patients randomized to the filgotinib induction were re-randomized to filgotinib induction dose maintenance arm or placebo arm (Peyrin-Biroulet et al., 2020). The composite primary endpoint of EBS remission at week 58 was achieved in 37.2% in the filgotinib 200 mg group vs. 11.2% in the placebo arm (*p* < 0.025) and 23.8% in the filgotinib 100 mg group vs. 13.5% in the placebo arm (*p* < 0.05) (Peyrin-Biroulet et al., 2020). Overall, filgotinib was well tolerated. Two cases of venous thromboembolism were reported in the placebo arm but none in the filgotinib group. Herpes zoster was reported in two patients in the filgotinib arms (one in each of the filgotinib groups) but none in the placebo groups (Peyrin-Biroulet et al., 2020).

A phase III clinical trial of filgotinib in CD is currently in progress (NCT02914561; NCT02914600). In addition, a phase II study evaluating filgotinib in perianal fistulizing CD is ongoing (NCT03077412).

#### Upadacitinib

Upadacitinib is an oral JAK inhibitor that has high selectivity for JAK 1 inhibition. It is currently approved for the treatment of rheumatoid arthritis with moderate to severe activity who have either failed or are intolerant to methotrexate. In a phase II clinical trial (CELEST), patients with moderate to severe CD (n = 220) who failed immunosuppressants or biologics were assigned to either placebo or upadacitinib at the following doses: 3 mg twice a day (BID), 6 mg BID, 12 mg BID, 24 mg BID for 16-weeks induction (Sandborn et al., 2020c). Post-induction, patients were re-randomized to upadacitinib at the following doses: 3 mg BID, 12 mg BID, 24 mg once daily (QD) for 36 weeks (maintenance). Although clinical remission at week 16 was not significantly different between the treatment groups and placebo, endoscopic remission at week 12/16 was significantly higher in the upadacitinib groups compared to placebo (Sandborn et al., 2020c). Higher doses of upadacitinib were associated with higher rates of endoscopic remission in this trial.

In the phase II UC-ACHIEVE trial, moderate to severe UC patients (n = 250) who failed or were intolerant to immunosuppressive or biologic therapy were assigned to placebo or upadacitinib at the following once daily doses: 7.5, 15, 30, 45 mg (Sandborn et al., 2020e). Clinical remission was significantly higher in the 15 mg (14.3%; *p* = 0.013), 30 mg (13.5%; *p* = 0.011), and the 45 mg groups (19.6%; *p* = 0.002) compared to 0% in the placebo group. Similarly, endoscopic improvement at week 8 (Mayo endoscopic subscore ≤1) was significantly higher in a dose dependent manner in the upadacitinib groups compared to placebo (Sandborn et al., 2020e).

In terms of safety, there were a total of three cases of herpes zoster in the upadacitinib group compared to none in the placebo group in the CELEST trial and 1 case in the upadacitinib group compared to none in the placebo group in the UC-ACHIEVE trial (Sandborn et al., 2020c; Sandborn et al., 2020e). Once case of thromboembolism occurred in a patient on the 45 mg upadacitinib dose in the UC-ACHIEVE trial.

Currently, upadacitinib is in phase III clinical trials for CD (NCT03345836, NCT03345823) and UC (NCT03653026, NCT03006068, NCT02819635).

#### TD-1473

TD-1473 is an oral pan-JAK inhibitor that is designed to be gut specific to limit systemic toxicity of pan-JAK inhibition. In a phase 1b trial, patients with moderate to severe UC (n = 40) were assigned to placebo or one of the following once daily TD-1473 doses: 20 mg, 80 mg or 270 mg) for 28 days (Sandborn et al., 2020f). Only five of the 40 patients were exposed to anti-TNF therapies in the past. Clinical response rate was 20% in the 20 and 80 mg groups compared to 55% in the 270 mg group while the clinical response rate was 11% in the placebo group (no statistical analyses performed as study not powered for outcomes) (Sandborn et al., 2020f). There were also trends in endoscopic improvement along with reduction in fecal calprotectin and c-reactive protein (CRP) levels with TD-1473. Overall adverse event rates were similar in the placebo group at 44.4 and 38.7% in the TD-1473 groups. Phase IIb/III studies of TD-1473 for moderate to severe UC are currently recruiting (NCT03758443, NCT03920254). In addition, a phase II study of TD-1473 in moderate to severe CD in underway (NCT03635112).

#### TYK2 Inhibitors

TYK2 is one of the JAK-STAT family proteins. It is involved in intracellular cytokine signaling. Brepocitinib (PF-06700841) is an oral TYK2/JAK1 inhibitor that has been shown to be well tolerated and effective in phase I and phase IIa studies of patients with plaque psoriasis (Banfield et al., 2018; Forman et al., 2020). PF-06651600 is an oral selective JAK3 inhibitor. Phase IIa/IIb trials of combination brepocitinib (PF-06700841) and PF-06651600 are currently recruiting for moderate to severe CD (NCT03395184) and UC (NCT02958865).

BMS-986165 is an oral selective TYK2 inhibitor. Phase II trials of BMS-986165 in CD (NCT03599622) and UC (NCT03934216) are currently recruiting.

### IL-12/IL-23 Inhibitors

IL-23 is a regulator of T-helper (Th)-17 cell and type 3 innate lymphoid cell (ILC3) pathways that lead to inflammatory cytokine production and inflammation and polymorphisms of the IL-23 receptor gene may be associated with increased susceptibility to CD (Feagan et al., 2017). IL-23 prevents regulatory T-cell response in the intestine, and therefore increases inflammation in this gut (Feagan et al., 2017; Sands et al., 2017). Ustekinumab, which inhibits both IL-12 and the IL-23 p40 subunit, has been shown to be effective for induction and maintenance of remission in patients with moderate-severe CD and UC (Feagan et al., 2016; Sands et al., 2019a).

However, it is unclear if the effect of ustekinumab is mainly driven by its inhibition of IL-12, IL-23 or both (Deepak and Sandborn, 2017). Selective inhibition of the IL-23 pathway, which leaves the IL-12 pathway intact, carries the potential advantage of less severe infection and decreased malignancy risk (Deepak and Sandborn, 2017; Baker and Isaacs, 2018). A potential benefit of only blocking the p19 subunit of IL-23 is possible increased safety since the IL-12 mediated Th1 response, which is required in the immune response to intracellular pathogens, is left intact (Deepak and Sandborn, 2017).

#### Risankizumab

Risankizumab is a humanized monoclonal antibody against the p19 subunit of IL-23. The induction dosing is provided intravenously (IV) while the maintenance dosing is a subcutaneous (SC) injection. A phase II trial of induction therapy with risankizumab was conducted in patients with moderate-severe CD; 93% of patients were previously exposed to at least one anti-TNF agent (Deepak and Sandborn, 2017; Feagan et al., 2017). Risankizumab (200 mg or 600 mg IV) was compared to placebo at weeks 0, 4, and 8. The primary outcome was clinical remission (defined as a CDAI <150) at week 12 (Feagan et al., 2017).

At week 12, those treated with risankizumab had significantly higher rates of clinical remission vs. placebo (30.5 vs. 15.4%, *p* = 0.0489), clinical response (39 vs. 20.5%, 0 = 0.0273), endoscopic remission (17 vs. 3%, *p* = 0.0015), and deep remission (7 vs. 0, *p* = 0.0107) (Feagan et al., 2017). There was no difference in the proportion of mucosal healing between those treated with risankizumab (200 mg: 2%, 600 mg 7%) vs. placebo (3%) (200 mg v. placebo, *p* = 0.97 and 600 mg v. placebo, *p* = 0.31) (Feagan et al., 2017). Rates of adverse events (AEs) were similar between those treated with placebo and risankizumab. The most common AE was worsening of underlying CD (Feagan et al., 2017). Other AEs included nausea, abdominal pain, arthralgia, and headache (Feagan et al., 2017). There were no dose-related increases in AE in those treated with risankizumab (Feagan et al., 2017).

An open-label extension (OLE) study was completed to study the efficacy and safety of extended IV induction and SC maintenance therapy (Feagan et al., 2018). Those who did not achieve deep remission in the 12-weeks induction phase of the previously described phase II induction study received open-label IV risankizumab 600 mg every 4 weeks for 12 weeks; patients who were in clinical remission at week 26 were included in the maintenance phase and received open-label SC risankizumab 180 mg every 8 weeks for 26 weeks (Feagan et al., 2018). The following definitions were used: clinical remission (CDAI <150), endoscopic response (>50% Crohn’s disease Endoscopic Index of Severity [CDEIS] reduction from baseline), endoscopic remission (CDEIS ≤4, or ≤2 for patients with isolated ileitis) (Feagan et al., 2018). One-hundred and one patients received the extended induction dose, and 53% (54/101 patients) were in clinical remission at week 26 (Feagan et al., 2018). Sixty-two patients received risankizumab maintenance therapy, and at week 52 clinical remission was maintained in 71% (44/62 patients), 81% (50/62) had clinical response, 35% (22/62) were in endoscopic remission, 24% (15/62) had mucosal healing, and 29% (18/62) achieved deep remission (Feagan et al., 2018). There were no new safety signals noted and the most frequent AEs included arthralgia, headache, abdominal pain, nasopharyngitis, nausea, and pyrexia (Feagan et al., 2018).

The long-term safety of risankizumab was studied in the phase 2 OLE study and no new safety signals were noted; 92% of patients reported AEs and 35% of patients experienced serious adverse events (Ferrante et al., 2020). The most common AEs included nasopharygnitis (31%), gastroenteritis (23%), and fatigue (20%) (Ferrante et al., 2020). Nine percent of patients had serious infections while 5% of patients reported opportunistic infections. There were no malignancies or deaths (Ferrante et al., 2020).

Overall, risankizumab was well-tolerated and found to be effective compared to placebo in inducing clinical remission, response, and endoscopic remission at week 12. Currently, we are awaiting results from phase III clinical trials (NCT03105128; estimated study completion date February 2021). In addition, a phase II/III clinical trial of risankizumab in ulcerative colitis is currently recruiting (NCT03398148).

#### Brazikumab (MEDI 2070)

Brazikumab, a human immunoglobulin G2 monoclonal antibody, selectively binds the p19 subunit of IL-23 (Sands et al., 2017). Like risankizumab, the induction dose is an IV infusion while the maintenance dose is a SC injection. A phase IIa trial included 119 patients with moderate-severe CD who either failed or were intolerant to anti-TNF agents; 31% of patients were exposed to at least one anti-TNF. Patients were given either brazikumab 700 mg IV or placebo at weeks 0 and 4, and then received open label brazikumab 210 mg subcutaneously every 4 weeks from weeks 12 to 112 (Sands et al., 2017). The primary outcome was clinical response (CDAI decrease of 100 points from baseline or clinical remission [CDAI <150]) at week 8 (Sands et al., 2017). Compared to placebo, a higher proportion of patients treated with brazikumab achieved clinical response at week 8 (49.2% v. 26.7%, *p* = 0.01) as well as a composite outcome of clinical response and 50% reduction from baseline in either fecal calprotectin or CRP concentration (42.4% v. 10%, *p* < 0.001) (Sands et al., 2017). There was no difference in clinical remission at week eight between those treated with brazikumab vs. placebo (27.1 vs. 15%, *p* = 0.10) (Sands et al., 2017). A similar proportion of patients who received placebo and active treatment during the initial double-blind period achieved clinical response, clinical remission, the composite outcome of clinical response and at least 50% reduction from baseline in either fecal calprotectin or CRP concentration at week 24 (57.7 vs. 53.8%, 40.4 vs. 42.3%, and 48.1 vs. 46.2%, respectively) (Sands et al., 2017). Patients with higher baseline serum concentration of IL-22 were found to be more likely to responds to brazikumab than to placebo (Sands et al., 2017; Sabino et al., 2019).

There was no difference in proportions of patients with treatment-emergent AEs or serious AEs between placebo and brazikumab treated groups (Sands et al., 2017). Equal numbers of clinically significant infections occurred in patients receiving brazikumab in both study periods and in patients receiving placebo in the double-blind period (Sands et al., 2017). The most common AEs included headache, nasopharyngitis, and abdominal pain (Sands et al., 2017).

In summary, two infusions of brazikumab at weeks 0 and 4 achieved a higher rate of clinical response at week 8 compared to placebo and continued SC dosing every 4 weeks led to ongoing clinical response and remission at week 24 (Sands et al., 2017). Brazikumab was generally well-tolerated in this study with follow-up to 24 weeks (Sands et al., 2017). Of note, there was no endoscopic evaluation or imaging as part of this phase 2a study (Sands et al., 2017). Currently, brazikumab is in a phase IIb/III trial in patients with CD (NCT03759288; anticipated completion date Dec 2022) and is in a phase II/open label extension in UC patients (NCT03616821; anticipated completion date april 2023).

#### Mirikizumab

Mirikizumab is an IV and SC immunoglobulin G4-variant monoclonal antibody, and it binds to the p19 subunit of IL23 (Sandborn et al., 2020d). A phase II study of 249 patients with moderate-severe UC was conducted to assess the efficacy and safety of mirikizumab; 63% of patients were previously exposed to a biologic (Sandborn et al., 2020d). Patients were randomized to either IV placebo, mirikizumab 50 mg, or 200 mg, or mirikizumab 600 mg at weeks 0, 4, and 8 (Sandborn et al., 2020d). Those with clinical response to mirikizumab at week 12 were then randomized to maintenance mirikizumab 200 mg every 4 weeks or every 12 weeks (Sandborn et al., 2020d). The primary endpoint was clinical remission (Mayo rectal bleeding subscore 0, with 1-point decrease from baseline for stool frequency, and 0 or one for endoscopy) (Sandborn et al., 2020d). Clinical remission at week 12 is as follows: 15.9% (95% CI, 6.8–24.9; *p* = 0.66) in the mirikizumab 50 mg group; 22.6% (12.2–33.0; *p* = 0.004) in the mirikizumab 200 mg group; and 11.5% (3.5–19.5, *p* = 0.142) in the 600 mg group vs. 4.8% (0–10) in the placebo groups (Sandborn et al., 2020d). Endoscopic improvement at week 12 was seen in 23.8% (13.3–34.3, *p* = 0.12) of the mirikizumab 50 mg group; 30.6% (23.3–34.3; *p* = 0.0007) in the 200 mg group, and 13.1% (4.6–21.6; *p* = 0.215) in the 600 mg group vs. 6.3% (0.3–12.4) in the placebo treated groups (Sandborn et al., 2020d).

Following the 12 weeks induction period, 93 mirikizumab treated patients achieved clinical response and were randomized to SC mirikizumab at 200 mg every 4 weeks or every 12 weeks (Sandborn et al., 2020d). At week 52, 53.7 and 39.7% of patients treated every 4 weeks and every 12 weeks, achieved clinical remission, respectively (Sandborn et al., 2020d). These rates were similar in those who were both biologic-exposed and biologic-naïve (Sandborn et al., 2020d). Endoscopic remission (Mayo endoscopic subscore = 0) at week 52 were 14.8% (every 4 weeks) and 28.3% (every 12 weeks) (Sandborn et al., 2020d). Durable clinical remission (clinical remission at both weeks 12 and 52) was 61.1% in the every 4 weeks maintenance dose and 38.5% in the every 12 weeks group (Sandborn et al., 2020d).

The most frequent AEs included nasopharyngitis, worsening of UC, headache, anemia, cough, nausea; no dose related increases in AEs were reported (Sandborn et al., 2020d).

An open label extension of this phase II study looked at the outcome of patients treated with mirikizumab who did not respond clinically in the initial induction period (Sandborn et al., 2020a). These patients were randomized to either 600 mg IV mirikizumab or 1,000 mg IV mirikizumab every 4 weeks (Sandborn et al., 2020a). At week 24, those who had a clinical response continued the maintenance period of 200 mg SC mirikizumab (Sandborn et al., 2020a). Endpoints included clinical remission (Mayo rectal bleeding = 0, 0 or 1 with a 1 point decrease from baseline), clinical response, endoscopic remission (Mayo endoscopic subscore = 0), or endoscopic improvement (endoscopic subscore of 0 or 1) at weeks 24 or 52 (Sandborn et al., 2020a). In the 12 weeks extension of the mirikizumab 600 mg dose and 1,000 mg mirikizumab dose, 50 and 43.8%, respectively, achieved clinical response and 15 and 9.4%, respectively, achieved clinical remission (Sandborn et al., 2020a). Endoscopic improvement was seen in 20% of patients in the 600 mg group and 15.6% of patients in the 1,000 mg group (Sandborn et al., 2020a). In those who had clinical response at week 24 and continued to maintenance therapy, 65.8% remained in clinical response, 26.3% had clinical remission, and 34.2% had endoscopic improvement at week 52 (Sandborn et al., 2020a). No new AEs identified.

In summary, the phase II dose ranging study of mirikizumab showed a trend toward inducing clinical remission and response around week 12 in patients with moderate-severe UC (Sandborn et al., 2020d). In those who did not initially respond to induction dosing, extended IV dosing of mirikizumab for 12 additional weeks showed a clinical response in about 50% of patients (Sandborn et al., 2020a).

Given its efficacy in patients with UC, mirikizumab was studied for its safety and efficacy in patients with CD in a phase II study (Sands et al., 2019b). Patients were randomized to mirkizumab 200 mg, 600 mg, 1,000 mg and placebo and this was administered at weeks 0, 4, and 8 (Sands et al., 2019b). The primary endpoint was endoscopic response (50% reduction from baseline in SES-CD) at week 12 (Sands et al., 2019b). Endoscopic response rates were higher in the mirkizumab groups compared to placebo at week 12 [200 mg: 25.8% (95% CI: 10.4–41.2), *p* = 0.079; 600 mg: 37.5% (20.7–54.3), *p* = 0.003; 1,000 mg: 43.8 (31.6–55.9), *p* < 0.001; placebo: 10.9 (3.3–18.6)] (Sands et al., 2019b). Additionally, patient reported outcome remission rates and CDAI response rates were greater in the 600 and 1000 mg mirikizumab group compared to placebo (Sands et al., 2019b). The frequencies of serious AEs and treatment-emergent AEs were similar between placebo and treatment groups (Sands et al., 2019b).

Mirikizumab was also evaluated for long-term efficacy and safety over 52 weeks in the phase II maintenance SERENITY study (Sands et al., 2020). Patients who achieved ≥1 point improvement in SES-CD at week 12 on mirikizumab were then re-randomized to maintenance mirikizumab IV or SC every 4 weeks (Sands et al., 2020). The primary endpoints were CDAI remission (<150 points), patient reported outcome (PRO) remission (stool frequency ≤2.5 and abdominal pain ≤1 and not worse than baseline), endoscopic response (50% reduction from baseline in SES-CD), and endoscopic remission (SES-CD score <4 for ileal-colonic disease or <2 for isolated ileal disease, and no subscore >1) (Sands et al., 2020). Endoscopic response rates were 58.5 and 58.7%; PRO remission rates were 46.3 and 45.7% in the IV and SC groups, respectively (Sands et al., 2020). In those who achieved endoscopic response at week 12, 69.6 and 66.7% had an endoscopic response at week 52 in the IV and SC groups, respectively (Sands et al., 2020). Also, in those who had endoscopic remission at week 12, 50 and 64.3% maintained endoscopic remission at week 52, respectively, in the IV and SC groups (Sands et al., 2020). There were similar treatment-emergent AEs and serious AEs in both the IV and SC groups (Sands et al., 2020).

Treatment with mirikizumab did have a dose-dependent improvement in endoscopic response and remission as well as patient-reported response and remission at week 12 in patients with CD (Authors, 2019) (A SPECIAL MEETING REVIEW EDITION: Highlights in Inflammatory Bowel Disease From the 14th Congress of ECCO: A Review of Selected Presentations From the 14th Congress of the European Crohn's and Colitis Organization (ECCO) * March 6–9, 2019 * Copenhagen, DenmarkSpecial Reporting on:* VARSITY: A Double-Blind, Double-Dummy, Randomized Controlled Trial of Vedolizumab vs. Adalimumab in Patients With Active Ulcerative Colitis* Analyses of Data From the VISIBLE 1 and 2 Trials: Vedolizumab in Patients With Ulcerative Colitis or Crohn's Disease* Improved Endoscopic Outcomes and Mucosal Healing of Upadacitinib as an Induction Therapy in Adults With Moderately to Severely Active Ulcerative Colitis: Data From the U-ACHIEVE Study* Long-Term Safety of Vedolizumab in Ulcerative Colitis and Crohn's Disease: Final Results From the GEMINI LTS Study* Pediatric Crohn's Disease Adalimumab Level-Based Optimization Treatment (PAILOT) Randomized Controlled Trial* Maintenance Treatment With Mirikizumab, a P19-Directed IL-23 Antibody: 52-Week Results in Patients With Moderately to Severely Active Ulcerative Colitis* Real-World Effectiveness and Safety of Vedolizumab and Anti-TNF Therapy in Biologic-Naive Patients With Ulcerative Colitis or Crohn's disease: Results From the EVOLVE Study* A Randomized, Multicenter, Double-Blind, Placebo-Controlled Study of a Targeted-Release Oral Cyclosporine Formulation in the Treatment of Mild to Moderate Ulcerative Colitis: Efficacy Results* Real-World Analyses of Patients With IBD Treated With VedolizumabPLUS Meeting Abstract Summaries With Expert Commentary by: Edward V. Loftus Jr, MD Professor of Medicine Division of Gastroenterology and Hepatology Mayo Clinic Rochester, Minnesota, 2019). Mirikizumab also had been shown to have sustained efficacy to week 52 (Sands et al., 2020). Mirkizumab for CD is currently being studied in a phase III trial (NCT03926130). Phase III Induction, maintenance and open label extension studies are currently recruiting for UC (NCT03518086, NCT03524092, NCT03519945).

#### Guselkumab

Guselkumab is an IV and SC anti-IL23 that is currently approved for the treatment of moderate-severe plaque psoriasis (Sandborn et al., 2020i). GALAXI 1 is a phase II study that evaluated the efficacy and safety of guselkumab in patients with moderate-severe CD who either had intolerance or inadequate response to corticosteroids, immunosuppressive agents and/or biologics (Sandborn et al., 2020i). Patients were randomized to guselkumab 200 mg, 600 mg, or 1,200 mg at weeks 0, 4, and 8; ustekinumab (the reference arm) ∼6 mg/kg IV at week 0 and 90 mg SC at week 8; or placebo IV (Sandborn et al., 2020i). Outcomes of interest included change in CDAI scores compared to baseline, clinical remission (CDAI < 150) and response, clinical biomarker response (clinical response and ≥50% reduction from baseline in CRP or fecal calprotectin), endoscopic response, and safety (Sandborn et al., 2020i). Two-hundred and fifty CD patients were included in this study and about 50% had failed biologics (Sandborn et al., 2020i). There were significant decreases of CDAI from baseline in all guselkumab groups compared to placebo at week 12 (Sandborn et al., 2020i). Compared to placebo, there was a higher proportion patients in all guselkumab groups who achieved clinical remission (200 mg: 54%, 600 mg: 56%, 1,200 mg: 50%, placebo: 15.7%), clinical biomarker response (200 mg: 54%, 600 mg: 48%, 1,200 mg: 42%, placebo: 11.8%), and endoscopic response (200 mg: 36%, 600 mg: 40%, 1,200 mg: 36%, placebo: 11.8%) (Sandborn et al., 2020i). In those who failed biologics, 45.5% (35/77) in the guselkumab group and 12.5% (3/24) in the placebo group achieved clinical remission at week 12 (Sandborn et al., 2020i). The rates of AE, infections, and serious AE were similar across all guselkumab treatment arms compared to placebo (Sandborn et al., 2020i).

In summary, in patients with moderate-severe CD, guselkumab was more efficacious in terms of clinical response, remission, endoscopic response, and clinical-biomarker response compared to placebo. There was no clear dose-response in this phase II study (Sandborn et al., 2020i). Guselkumab Is currently in phase II/III trials with an estimated study completion date in October 2024 (NCT03466411).

Additionally, a phase IIb/III study of the safety and efficacy of guselkumab in patients with moderate-severe UC was started in September 2019 (NCT04033445; anticipated completion by July 2025). In addition, a phase IIa study is evaluating combination treatment of guselkumab and golimumab in moderate-severe UC (NCT03662542) (Hanzel and D'Haens, 2020).

### IL-6 Inhibitors

#### PF-04236921

PF-04236921 is a fully human immunoglobulin G2 monoclonal antibody that binds IL-6, which has many pro-inflammatory effects. A phase II study (ANDANTE I) aimed to evaluate the efficacy and safety of PF-04236921 in patients with moderate-to-severe CD who had inadequately responded to anti-TNF therapy (Danese et al., 2019). This study was then followed by an open-label extension study (ANDANTE II) with the primary aim of studying long-term safety, tolerability, and immunogenicity. For the phase II study, patients were randomized 1:1:1:1 to receive placebo or PF-04236921 10, 50, or 200 mg SC on days 1 and 28. The primary endpoint for the induction study was CDAI-70 response at weeks 8 or 12. Secondary endpoints included CDAI-70 and CDAI-100 response rates, CDAI remission (CDAI score <150). The primary aim of the OLE study was safety (Danese et al., 2019).

In the induction study PF-04236921 50 mg was found to have significantly higher CDAI-70 rates compared to placebo at weeks 8 (49.3 vs. 30.6%, *p* < 0.05) and 12 (47.4 vs. 28.6%, *p* < 0.05) (Danese et al., 2019). CDAI remission rates with PF-04236921 50 mg daily were significantly higher at week 12 compared to placebo 27.4 vs. 10.9%, *p* < 0.05) (Danese et al., 2019). The PF-04236921 10 mg dose did not meet the primary end point.

One-hundred and nine one patients entered the OLE study, and 89 (46%) were CDAI responders at OLE baseline. A majority of patients (77.8%) had their PF-04236921 dose escalated to 100 mg between weeks 8 and 48. In those who had been on drug in the induction period (n = 65), the HBI response and remission rates were 40 and 32%, respectively, at week 48 (Danese et al., 2019). It was rare to find antidrug antibodies. One out of 680 serum samples (0.1%) were found to be positive for anti-drug antibody (at week 4 with dose of 50 mg). CRP levels were noted to be suppressed during the OLE treatment period.

The most common serious adverse effects with CD-related, such as worsening of CD and abdominal pain, and nasopharyngitis. In the OLE study, most AEs were mild-moderate in severity. One death occurred in the induction study, and this was in the 50 mg group; the patient died of respiratory failure secondary to pneumonia and was thought to be unrelated to the treatment. Cases of GI perforation and GI abscesses were seen in the induction and OLE studies of patients on PF-04236921; most cases were identified in areas of previous disease involvement or surgery.

In summary, in patients with moderate-severe CD, PF-04236921 50 mg was more efficacious than placebo in inducing response and remission at week 12. In those in the OLE, who were initially on active drug during the induction period, about 40% had an HBI response rate at week 48. There are signals of GI perforation and abscess in the PF-04236921 treatment groups, and therefore this signal will need to be carefully monitored in any future clinical trials. At this time there are no phase three trials of PF-04236921.

### Human IL-22Fc Fusion Protein

#### UTTR1147A

The activation of the IL-22 pathway may help to increase epithelial tight junctions, promote mucus production, and lead to secretion of antimicrobial peptides (Rothenberg et al., 2019). Ultimately this pathway may promote tissue regeneration, and therefore it is a valuable treatment target. UTTR1147A (IL-22Fc) is a fusion protein consisting of a linked human IL-22 and crystallizable fragment (Fc) of the human IgG4 (Rothenberg et al., 2019). A recent phase 1a trial studied single, ascending intravenous (1–120 μg/kg) and subcutaneous (3–120 μg/kg) doses of UTTR1147A on safety and tolerability in healthy volunteers (Rothenberg et al., 2019). The maximum tolerated IV dose was 90 μg/kg and most common AEs were dose-dependent but reversible skin effects. UTTR1147A increased proportionally to the doses given, and this caused elevations of IL-22 signaling biomarkers without causing systemic inflammation. Thus, preliminary data shows that UTTR1147A promotes the IL-22 signaling pathway and may lead to tissue regeneration without immunosuppression. This would be a novel mechanism of action in those with epithelial injury, such as what occurs in patients with IBD.

A phase Ib study analyzed UTTR1147A in healthy volunteers and UC patients. 38 healthy volunteers and 24 UC patients were given UTTR1147A or placebo at doses ranging between 30–90 μg/kg either weekly or monthly (6:2 UTTR1147A: placebo per cohort) (Wagner et al., 2020). The Mayo Clinic score was evaluated at baseline, day 30, and day 85. The study found that UTTR1147A was safe and well tolerated in both groups of patients and most common side effects were dermatologic effects of dry skin, erythema, and pruritis. Interestingly, at the same dose level, UC patients had relatively lower drug exposures than healthy volunteers and this was thought to be due to faster drug clearance (Wagner et al., 2020). Additionally, UC patients had attenuated pharmacodynamics response (production of biomarkers such as CRP) compared to healthy volunteers (Wagner et al., 2020). However, this study showed that there was an adequate safety and PK profile in both cohorts, and UTTR1147A did produce pharmacodynamics biomarkers in both cohorts. Further data would be important to study this novel mechanism of action in the treatment of patients with IBD.

### Anti-Adhesion Molecules

Migration of pro-inflammatory T cells into the gut facilitates inflammation which is characteristic of CD and UC (Targan et al., 2007; Lin et al., 2015; Shivashankar and Pardi, 2017b). Interaction between surface-expressed α4β1 and α4β7 integrins on lymphocytes and adhesion molecules [vascular cell adhesion molecule-1 (VCAM-1) or mucosal addressin cell adhesion molecule-1 (MAdCAM-1)], which are present on endothelial cells, allow for activated effector T cells to target the gut (Shivashankar and Pardi, 2017b). This interaction allows for movement of T cells out of the blood stream and into the gastrointestinal tract (Sugiura et al., 2013; Shivashankar and Pardi, 2017b; Sandborn et al., 2020b). Natalizumab (NAT), a humanized IgG4 monoclonal antibody that targets the α4-integrin subunit of α4β1 and α4β7 on the surface of lymphocytes, has been approved for CD and multiple sclerosis since it blocks lymphocyte trafficking to the gut and brain. NAT has been associated with progressive multifocal leukoencephalopathy (PML) (Feagan et al., 2013; Park and Jeen, 2018). On the other hand, vedolizumab (VDZ) is a humanized IgG1 monoclonal antibody that specifically targets the α4β7 integrin, and it has been approved for UC and CD and therefore selectively prevents leukocyte migration to the gut (Feagan et al., 2013; Park and Jeen, 2018). In this section, we will focus on novel anti-adhesion molecules and a novel method of administration of VDZ.

#### SC Vedolizumab

VDZ, an infusion medication, was approved in 2014 for moderate-severe UC and CD. From clinical trial data, CD patients who responded to induction dosing at week 6 were more likely to achieve clinical remission, clinical response, and corticosteroid-free remission at week 52 compared to placebo (Sandborn et al., 2013). In addition, UC patients treated with VDZ in clinical trials had a significantly higher rate of clinical remission, clinical response, mucosal healing, and corticosteroid-free remission at week 52 compared to placebo (Feagan et al., 2013).

A new SC formulation of VDZ has been studied in those with UC; the benefit of this formulation is its convenient route of administration for patients who would like to avoid maintenance IV infusion therapy. A phase III open-label study (VISIBLE 1) of patients with moderate-severely active UC was conducted to assess the efficacy of SC VDZ as maintenance therapy (Sandborn et al., 2020b). The primary endpoint was clinical remission (total Mayo score ≤2 and no subscore >1) at week 52. VDZ 300 mg IV was administered at weeks 0 and 2 (Sandborn et al., 2020b). Those with clinical response at week 6 were then randomized to maintenance treatment with SC VDZ 108 mg every 2 weeks, IV VDZ 300 mg every 8 weeks, or placebo (Sandborn et al., 2020b). SC VDZ had a higher rate of clinical remission at week 52 compared to placebo in both anti-TNF naïve and anti-TNF exposed patients (53.7 vs. 18.9%, *p* < 0.01 and 33.3 vs. 5.3%, *p* = 0.023, respectively) (Sandborn et al., 2020b). There were also significantly higher rates of endoscopic improvement (56.6 vs. 21.4%, *p* < 0.001) and durable clinical response (64.2 and 28.6%, *p* < 0.001) when SC VDZ was compared to placebo (Sandborn et al., 2020b). There was no significant difference between SC VDZ and placebo in terms of durable clinical remission (15.1 vs. 5.4%, *p* = 0.076) or corticosteroid-free remission (28.9 vs. 8.3%, *p* = 0.067) (Sandborn et al., 2020b). Endpoints were generally similar between SC VDZ and IV VDZ.

There were no new safety signals when SC VDZ was compared to IV VDZ. The most common AE was worsening of UC disease activity, nasopharyngitis, upper respiratory infections, and anemia (Sandborn et al., 2020b). Eleven patients (10.4%) receiving SC VDZ had injection-site reactions characterized by rash, swelling, erythema, and pruritis (Sandborn et al., 2020b). There were no cases of PML or deaths. The VDZ pharmacokinetics achieved with SC VDZ was similar to that of the IV formulation (Sandborn et al., 2020b).

Overall, this study showed that SC vedolizumab was effective and well-tolerated as maintenance therapy for UC following an induction with IV VDZ. Efficacy outcomes were generally similar between maintenance SC VDZ and IV VDZ. Also, the safety profile was encouraging, and no new safety signals were identified. SC VDZ will give patients an option for an alternate mode of administration if they prefer to avoid long-term infusions. SC VDZ for moderate-severe UC will potentially be available in 2022.

#### Etrolizumab

While VDZ targets only the α4β7 integrin, etrolizumab is a SC humanized monoclonal anti-integrin antibody that selectively binds the β7 subunit of both α4β7 and αEβ7 integrins (Vermeire et al., 2014; Sandborn et al., 2020h). Therefore, etrolizumab controls movement of inflammatory cells into the gastrointestinal tract and also mediates inflammatory effects on the gut mucosa (Vermeire et al., 2014; Sandborn et al., 2020h). The phase II EUCALYPTUS study showed that etrolizumab was beneficial over placebo in patients with moderate-severe UC (Vermeire et al., 2014). In this study, 124 patients were randomized to two doses of SC etrolizumab—either 100 mg at weeks 0, 4, and 8 with placebo at week 2; or 420 mg loading lose (LD) at week 0, followed by 300 mg at weeks 2, 4, and 8—or placebo (Vermeire et al., 2014). The primary endpoint was clinical remission at week 10 (Mayo clinic score ≤2 with no individual subscore >1). Compared to placebo (0 patients in clinical remission), etrolizumab (100 mg: 21%, *p* = 0.004; LD+ 300 mg: 10%, *p* = 0.048) treatment was found to lead to a higher proportion of patients in clinical remission at week 10 (Vermeire et al., 2014). Adverse events occurred at a similar frequency across all groups—61% of 100 mg etrolizumab group, 49% in the LD+ 300 mg group, and 72% in the placebo group. No patients developed PML.

Etrolizumab has an extremely active phase III clinical trial program and includes direct comparisons to infliximab and adalimumab. There are currently six randomized controlled trials and two open label extension studies. The aim of HIBISCUS I and II (identical induction trials of etrolizumab 105 mg vs. adalimumab and placebo), GARDENIA (maintenance study studying etrolizumab 105 mg vs. infliximab), and LAUREL (maintenance trial evaluating etrolizumab 105 mg against placebo) is to study UC patients who are anti-TNF naïve. HICKORY is a maintenance trial studying etrolizumab 105 mg vs. placebo in anti-TNF exposed UC patients (Sandborn et al., 2020h). The primary endpoints are based on clinical response (≥3 point decrease and 30% reduction in Mayo clinic score and ≥1 point decrease in rectal bleeding or an absolute rectal bleeding score of 0 or 1), remission (MCS ≤2, with individual subscore ≤1 and rectal bleeding score of 0) at each trial’s specified time points (Sandborn et al., 2020h). COTTONWOOD is the open-label extension and safety monitoring study of UC patients treated with etrolizumab who were followed until week 108 (Sandborn et al., 2020h).

Early results from the HICKORY study included 130 UC patients and 45% had previously been exposed to ≥2 anti-TNFs (Peyrin-Biroulet et al., 2017). Stool frequency rates improved from baseline to week 4 in 30% and at week 14 in 50% of patients (Peyrin-Biroulet et al., 2017). Rectal bleeding remission rates were observed from baseline to week 4 in about 30% of patients and at week 14 in about 50% of patients (Peyrin-Biroulet et al., 2017). Biomarkers decreased at week 14 (fecal calprotectin: mean 57% decrease; CRP: 33% decrease) (Peyrin-Biroulet et al., 2017). Therefore, in patients previously exposed to anti-TNFs, etrolizumab led to symptom improvement as early as week 4 (Peyrin-Biroulet et al., 2017).

For patients with CD, the BERGAMOT study is an induction and maintenance trial evaluating anti-TNF naïve and experienced patients (etrolizumab 105 and 210 mg vs. placebo) (Sandborn et al., 2020h). Eligible patients may enter JUNIPER study, which will be the open label extension of etrolizumab 105 mg (Sandborn et al., 2020h). The co-primary endpoint for BERGAMOT is clinical remission (unweighted abdominal pain score ≤1 and stool frequency ≤3) and endoscopic improvement (>50% reduction in SES-CD from baseline) at weeks 14 and 62 (Sandborn et al., 2020h). The primary outcome for JUNIPER is long term efficacy (clinical remission assessed at 12 weeks intervals), endoscopic remission (SES-CD ≤4 [≤2 for pts with ileal disease] with no segment >1) at week 108 and incidence and severity of AEs (Sandborn et al., 2020h).

The BERGAMOT induction cohort included 300 CD patients who were randomized to etrolizumab 105 mg SC every 4 weeks, 210 mg at weeks 0, 2, 4, 8, and 12, or placebo for a 14 weeks induction period (Selinger et al., 2018). Previous anti-TNF exposure was noted in 73% of patients (Selinger et al., 2018). Symptomatic remission was seen in a greater number of patients treated with etrolizumab [105 mg: 20.8% (90% confidence interval 15.4–27.5); 210 mg: 24.8 (18.9–31.8)] compared to placebo (11.9 [6.6–20.5]) at week 14 (Selinger et al., 2018). Similarly, more patients treated with etrolizumab (105 mg: 21 [15.6–27.8] and 210 mg: 17.4 [12.4–23.7] achieved endoscopic improvement compared to placebo (3.4 [1.1–9.7]) at week 14 (Selinger et al., 2018). AEs were comparable between etrolizumab and placebo; there were no cases of PML or death (Selinger et al., 2018).

In addition to providing further data on clinical response, remission, and endoscopic remission data for etrolizumab, these data may help clinicians decide which biologic is an ideal first-line agent for their patient (Sandborn et al., 2020h). The HIBISCUS I and II and GARDENIA studies will be the first phase III trials in UC to study the efficacy of a novel agent directly compared to another agent, i.e., infliximab and adalimumab (Sandborn et al., 2020h).

#### AJM300

α4 or α4β7 targets have been shown to be possible therapeutic targets in the treatment of IBD since suppression of these integrins have controlled development of colitis in an animal model (Sugiura et al., 2013). A variety of small molecule α4 integrin antagonists have been studied in clinical trials for conditions such as asthma and multiple sclerosis. AJM300 is an oral small molecule that targets α4 integrin, and it is classified as a phenylalanine derivative (Yoshimura et al., 2015). This small molecule’s duration of action is very short, within one day of last dose as described in a phase IIa study (Yoshimura et al., 2015). AJM300 has been shown to be effective in experimental colitis model in mice; this study showed that the active metabolite of AJM300 inhibited the binding of α4β1/α4β7 integrin-expressing cells to VCAM-1/MAdCAM-1 *in vitro* (Sugiura et al., 2013). Given the α4 blockade, there may be a potential risk for PML in AJM-300 therapy (Yoshimura et al., 2015).

A phase IIa study including 102 patients with moderate active UC was conducted to assess the primary outcome measure of clinical response (decrease in Mayo score of at least 3 points and decrease of at least 30% from baseline, with decrease in rectal bleeding subscore of at least 1 point or an absolute rectal bleeding score of 0 or 1) at week 8 (Yoshimura et al., 2015). Patients who had inadequate response or intolerance to mesalamine or corticosteroids were given AJM300 960 mg orally or placebo three times daily for 8 weeks (Yoshimura et al., 2015). Compared to placebo, those treated with AJM300 had higher rates of clinical response (62.7 vs. 25.5%, *p* = 0.0002), clinical remission (23.5 vs. 3.9%, *p* = 0.0099), and mucosal healing (58.8 vs. 29.4%, *p* = 0.0014) (Yoshimura et al., 2015).

There was a significant increase in peripheral leukocyte count as would be expected from an anti-α4 integrin; this increase was seen as early as week 2 (Yoshimura et al., 2015). However, this finding normalized the day after the last dose given at week 8 thereby suggesting a short duration of action (Yoshimura et al., 2015). The most common adverse events were nasopharyngitis and UC, with higher AEs seen with UC in the placebo group (Yoshimura et al., 2015). Overall, however, there were similar rates of AEs observed between the treatment and placebo arms (Sabino et al., 2019). There were no serious AEs observed, and infection, and PML were not seen in this study (Yoshimura et al., 2015).

In summary, AJM-300 was more effective than placebo in inducing clinical response, clinical remission, and mucosal healing in moderately active UC patients who had only been exposed to mesalamine or steroids in the past. However, the study was limited by a small sample size and a short study duration (8 weeks) (Yoshimura et al., 2015). Unfortunately, there were extremely low number of patients who were intolerant or inadequately responding to steroids in this study, and there was no information on exposure to immunomodulators and anti-TNFs (Yoshimura et al., 2015). AJM300 is currently in phase III trials for UC patients, and the estimated study completion date is February 2021 (NCT03531892).

#### Abrilumab (AMG 181or MEDI 7183)

Abrilumab is an injectable fully human monoclonal immunoglobulin G2 antibody that targets the α4β7 integrin and prevents interaction with MADCAM-1 (Sandborn et al., 2017a). Abrilumab has a high bioavailability after SC dosing and a long half-life (about 31 days) (Sandborn et al., 2019).

A phase IIb study in patients with CD who had inadequate or loss of response or intolerance to immunosuppressives, anti-TNFs, or corticosteroids were randomized to receive placebo or abrilumab (21 mg or 70 mg) SC on day 1, weeks 2 and 4, and then every 4 for 24 weeks, or one dose of abrilumab 210 mg SC on day 1 (Sandborn et al., 2017a). The primary endpoint was clinical remission (CDAI < 150) at week 8 (Sandborn et al., 2017a). There was no significant difference in clinical remission at week 8 between abrilumab at any dose when compared to placebo (Sandborn et al., 2017a). Of note, there were higher rates of clinical remission at week 12 in those treated with a single dose of abrilumab 210 mg when compared to placebo in those who previously failed anti-TNF therapy (Sandborn et al., 2017a). AE rates were similar among all groups through week 24, and there were no cases of PML or death (Sandborn et al., 2017a).

A phase II induction and sustained remission study of abrilumab was conducted in patients with active moderate-severe UC who did not respond or lost response to usual medical therapy (Sandborn et al., 2019). Patients were randomized to SC placebo or abrilumab (7, 21, 70 mg) on day 1, week 2, week 4, and then every 4 weeks thereafter until week 24 or a single dose of 210 mg abrilumab on day 1 followed by placebo at the same dosing scheduled noted previously until week 24 (Sandborn et al., 2019). An open-label phase of the study involved administration of abrilumab 210 mg once every 3 months (Sandborn et al., 2019). The primary endpoint was remission (total Mayo score ≤2 points with no individual sub-score >1 point) at week 8 (Sandborn et al., 2019). There were significantly higher odds of achieving clinical remission, clinical response, and mucosal healing at week 8 in those treated with abrilumab 70 and 210 mg compared to placebo (Sandborn et al., 2019). In those with prior anti-TNF failure, there were greater clinical remission rates at week 8 in the abrilumab 70 and 210 mg dose (Sandborn et al., 2019).

However, there were no clear benefits of abrilumab treatment at week 24—in this study, patients continued their initial treatment regardless of whether they responded after the induction period (Sandborn et al., 2019). The odds of sustained remission did increase in patients who continued to take multiple doses of abrilumab 70 mg every 4 weeks compared to placebo (Sandborn et al., 2019).

There were no differences in AEs across treatment groups through week 24. The most common AE were non-serious infections, gastrointestinal effects, headache, and arthralgia (Sandborn et al., 2019). The only serious AE noted in more than one patient in the abrilumab treated group was worsening of UC (Sandborn et al., 2019). There were no cases of PML or death. Ten patients (8.6%) in the placebo group and 9 (3.8%) in the abrilumab group reported neoplasms (Sandborn et al., 2019). A limitation of this phase II study of abrilumab in UC include its short duration; phase III studies are needed to assess the long-term response of the 70 mg dose after 24 weeks (Sandborn et al., 2019).

In conclusion, while the phase II study of abrilumab in CD failed to meet its primary endpoint, the study in UC patients showed efficacy at week 8 with higher rates of clinical remission, clinical response, and mucosal healing. Since there was no clear benefit of abrilumab at week 24 in UC patients, longer term phase III studies are required to better assess this finding. Phase 3 studies have not yet started.

#### PF-00547659 (SHP647)

PF-00547659 is a SC fully human monoclonal antibody that binds to MAdCAM, thereby decreasing lymphocyte trafficking to the gut and leading to decreased gastrointestinal inflammation (Sandborn et al., 2018). A phase II dose ranging study of PF-00547659 was conducted in patients with moderate-severe CD who have a history of failure or intolerance to immunosuppressives and/or anti-TNF therapy (Sandborn et al., 2018). This study included 265 patients who were randomized to PF-00547659 22.5, 75 mg, or 225 mg or placebo (Sandborn et al., 2018). The primary endpoint was a CDAI 70-point decrease from baseline at week eight or 12 (Sandborn et al., 2018). There was no significant difference in CDAI response between the treatment group and placebo at week 8 (22.5 mg: 52.7%, 75 mg: 60.1%, 225 mg: 62.7%) or 12 (22.5 mg: 62%, 75 mg: 64.7%, 225 mg: 57.5%); this was likely due to a high clinical response in the placebo group (47.7% at week 8 and 58.6% at week 12) (Sandborn et al., 2018).

In a post-hoc analysis, the proportions of patients who achieved clinical remission were higher in all PF-00547659 dose groups than in placebo at weeks 8 and 12 in those with higher inflammatory burden as evidenced by higher median high sensitivity CRP values (>5 mg/L or >18.8 mg/L) or higher baseline SES-CD scores (>17) at baseline (Sandborn et al., 2018). The incidence of AEs, serious AEs, and discontinuation was similar across all treatment groups.

The long-term safety and efficacy portion of the study followed patients for a further 6 months (D’Haens et al., 2018a). The most common AEs with treatment included nasopharyngitis (5.6%), arthralgia (6.0%), and headache (5.2%) (D’Haens et al., 2018a). There were two deaths (multiorgan failure after postoperative aspiration following resection of the terminal ileum and metastatic adenocarcinoma of unknown primary) however neither were thought to be drug related (D’Haens et al., 2018a). Harvey Bradshaw Index response rates were sustained over 6 months of treatment (D’Haens et al., 2018a).

A post-hoc analysis also looked at endoscopic outcomes following induction therapy with PF-00547659 (D′Haens et al., 2018b). Mean total SES-CD scores across four colon and one ileal segments were found to trend toward a decrease across all treatment arms (except for the 75 mg dose) (D′Haens et al., 2018b). The 22.5 mg dose showed the largest trend toward improvement in endoscopic disease severity (mean change from baseline to week 10 at 22.5 mg dose: 4.0; 75 mg: 0; 225 mg: −1; placebo: 1.5) (D′Haens et al., 2018b). However, no consistent association between clinical and endoscopic improvement was observed (D′Haens et al., 2018b).

In patients with moderate-severe UC, a phase II study that evaluated the efficacy and safety of PF-00547659 in 357 patients who had failed or were intolerant to at least one usual therapy found the drug to be superior to placebo (Vermeire et al., 2017a; Sabino et al., 2019). The primary endpoint was clinical remission (Mayo score ≤2 with no individual subscore >1 and rectal bleeding subscore ≤1) at week 12 (Vermeire et al., 2017a). The remission rates in those treated with PF-00547659 (7.5 mg: 11.3%; 22.5 mg: 16.7%; 75 mg: 15.5%; 225 mg: 5.7%) were higher than placebo (2.7%) at week 12; the 225 mg dosing did not achieve statistical significance compared to placebo (Vermeire et al., 2017a). Similar to the findings from the CD trial, PF-00547659 was overall well-tolerated (Vermeire et al., 2017a).

In conclusion, in patients with CD, PF00547659 was not significantly different compared to placebo in achieving clinical response at weeks 8 or 12 likely due to high placebo response rates (Sabino et al., 2019). On the other hand, three out of four doses of PF00547659 did achieve significantly higher rates of clinical remission than placebo in patents with UC. Phase III studies in patients with IBD have not yet been announced.

### Oral Anti-tumor Necrosis Factor Agents

Anti-TNF agents were the first class of biologics approved for the treatment of moderate to severe UC and CD. They have revolutionized the treatment paradigm of IBD and have proven to be effective (Sands et al., 2004; Rutgeerts et al., 2005; Hanauer et al., 2006; Sandborn et al., 2007; Schreiber et al., 2007). One of the limitations of current anti-TNF agents is that they are currently administered by IV or as SC injectables only. This is associated with risk of infusion/injection reactions, systemic side effects and increased cost of repeated infusions/injections. An oral and intestinally restricted anti-TNF agent would help overcome some of these challenges of current anti-TNF agents.

#### AVX-470

AVX-470 is an oral polyclonal immunoglobulin that is derived from the colostrum of cows that have been immunized with human recombinant anti-TNF. In the first human study, 36 patients with UC received either AVX-470 (pH dependent delayed release capsules) at doses of 0.2, 1.6, and 3.5 g/day or placebo (Harris et al., 2016). In terms of safety, AVX-470 was well tolerated and the total rate of AEs was 52% compared to 78% in the placebo group. At 4 weeks, the overall clinical response and remission rates were 25.9 and 3.7% in the AVX-470 group compared to 11.1 and 0% in the placebo group, respectively. Endoscopic remission was achieved in 7.4% in the AVX-470 group compared to none in the placebo group. In term of immunogenicity, no human anti-bovine antibodies were detected.

#### OPRX-106

OPRX-106 is an oral anti-TNF agent. It is a combination of plant cell-expressed human anti-TNF receptor II fused to IgG1 Fc domain. The plant cells function as a delivery mechanism. A phase II randomized, 2-arm, open label clinical trial of 25 patients with UC treated with OPRX-106 at 2 or 8 mg daily for 8 weeks was performed (Almon et al., 2020). A total of 18 patients completed the study. Clinical response defined as a decrease in the Mayo score by at least 3 points was achieved in 67% and clinical remission (Mayo score ≤2 without sub-score being greater than 1) was achieved in 28%. Mucosal healing (Mayo endoscopic score ≤1) was achieved in 33% (Almon et al., 2020). In terms of immunogenicity, no anti-drug antibodies were detected.

Oral anti-TNF agents have the potential of enhanced safety, decreased immunogenicity and higher patient acceptance. Although promising, the current available data on efficacy and safety of oral anti-TNF agents is limited and future larger trials are needed to evaluate efficacy, safety, feasibility and cost effectiveness.

### Sphingosine-1-Phosphate Receptor Modulators

Sphigosine-1-phosphate is a sphingolipid ligand of G protein coupled receptors (S1P1-S1P5) which are responsible for controlling the egress of lymphocytes from lymphoid organs. The S1P/S1PR interaction can also result in internalization of the S1PR which leads to decrease lymphocyte release from the lymphoid tissue into the circulation. Fingolimod was the first S1P receptor modulator approved for the treatment of multiple sclerosis in 2010 (Cohen et al., 2010; Kappos et al., 2010). Fingolimod is a non-selective S1P receptor modulator and has high affinity for S1PR 1 and S1PR3-5. Although overall well tolerated, fingolimod has been associated with adverse events such as bradycardia, hypertension, infection and elevated liver tests (Danese et al., 2018).

#### Ozanimod

Ozanimod is a S1P receptor modulator with selective affinity for S1PR1 and S1PR5. It was approved by the US FDA in 2020 for the treatment of relapsing MS (Cohen et al., 2016; Cohen et al., 2019; Comi et al., 2019).

TOUCHSTONE is a phase II double blind, randomized clinical trial of ozanimod 0.5 or 1 mg vs. placebo daily in moderate to severe UC (n = 197). The primary outcome was clinical remission at 8 weeks (Mayo clinic score ≤2; No sub-score > 1). The primary outcome was achieved in 16% in the ozanimod 1 mg group vs. 14% in the ozanimod 0.5 mg group vs. 6% in the placebo group (*p* = 0.048 and *p* = 0.14, respectively) (Sandborn et al., 2016). Mucosal healing at week 8 (defined as Mayo endoscopy sub-score ≤1) was achieved in 34% in the 1 mg group vs. 28% in the 0.5 mg group compared to 12% in the placebo group (*p* = 0.002 and *p* = 0.03, respectively). At week 32, clinical remission was 21% in the 1 mg group and 26% in the 0.5 mg group compared to 6% in the placebo group (*p* = 0.01 and *p* = 0.002, respectively). Mucosal and histologic healing rates were similarly significantly higher in the ozanimod groups compared to placebo at 32 weeks (Sandborn et al., 2016). In the ozanimod groups, one patient developed bradycardia (patient had previous history of bradycardia) and four patients developed elevated liver enzymes.

TRUE-NORTH is a phase III randomized, double-blind, placebo-controlled trial of induction and maintenance ozanimod in UC completed in 2020 (Danese et al., 2020a; Sandborn W. et al., 2020). In the induction phase of 10 weeks, moderately to severely active UC patients received either ozanimod 1 mg or placebo daily. The primary endpoint of clinical remission (based on 3-component Mayo score) was met in 18.4% in the ozanimod group vs. 6% in the placebo group (*p* < 0.0001) (Sandborn W. et al., 2020). Clinical remission was numerically higher then placebo but did not achieve statistical significance in the anti-TNF exposed patients who received ozanimod (10 vs. 4.6%; *p* = 0.195). Secondary outcomes of clinical response, endoscopic improvement and mucosal healing (both endoscopy and histology) rates were significantly in the total ozanimod group compared to placebo. The reported rate of bradycardia was 0.5% in the ozanimod group.

In the maintenance trial, 457 moderate to severe UC patients who achieved clinical response after 10 weeks of ozanimod induction therapy were then re-randomized at 1:1 to either ozanimod 1 mg or placebo and outcomes assessed at week 52 (Sandborn W. et al., 2020). Clinical remission (based on 3-component Mayo score) rates were significantly higher in the ozanimod group at 37% compared to 18.5% in the placebo group (*p* < 0.0001) (Danese et al., 2020a). Anti-TNF exposed patients who received ozanimod also had a significantly higher rate of clinical remission compared to placebo (28.9% vs. 10.1; *p* = 0.0053). All secondary endpoints of the trial were also met in the ozanimod group including: clinical response, endoscopic improvement, corticosteroid-free remission, maintenance of clinical remission, durable clinical remission and mucosal healing (both endoscopic and histologic) (Danese et al., 2020a). In this trial, ozanimod was also well tolerated with the most common reported AEs being abnormal liver tests and headaches. Elevation of alanine aminotransferase (ALT) was noted in 4.8% in the ozanimod group compared to 0.4% in the placebo group (Danese et al., 2020a).

For CD, ozanimod was evaluated in a phase II multicenter, uncontrolled, prospective observer-blinded endpoint trial, STEPSTONE (Feagan et al., 2020a). The trial had a 12-weeks induction period followed by a 100-weeks extension study. Patients with moderately to severely active CD (n = 69) received a 7-days dose escalation of ozanimod followed by 11 weeks of ozanimod 1 mg oral capsule daily. The primary endpoint was reduction in the Simple Endoscopic Score for Crohn’s disease (SES-CD) at 12 weeks from baseline. The mean change of SES-CD at week 12 was – 2.2 ± 6 and 23.2% of patients achieved endoscopic response (defined as SES-CD decrease by ≥ 50%) (Feagan et al., 2020a). Clinical remission (defined as CDAI <150) was noted in 39.1% of patients. The most common AE was CD flare and there were no cases of bradycardia or arrhythmias reported (Feagan et al., 2020a).

Phase III, placebo-controlled induction and maintenance studies of ozanimod in moderate to severe CD are currently recruiting (NCT03440372, NCT03440385, NCT03464097, NCT03467958).

#### Etrasimod

Etrasimod is an oral S1P receptor modulator with selectivity for S1PR1, S1PR4 and S1PR5. A phase II (OASIS; proof of concept), double-blinded trial randomized moderate to severe UC patients (n = 156) to either etrasimod 1 mg, etrasimod 2 mg or placebo in a 1:1:1 fashion for 12 weeks (Sandborn et al., 2020g). The primary endpoint was improvement in the modified Mayo Clinic score at 12 weeks from baseline and secondary endpoints included: the improvement in the total Mayo Clinic score, improvement in the two component Mayo Clinic score (rectal bleeding and endoscopy) and endoscopic improvement (endoscopy sub-score ≤ 1). The primary endpoint was achieved in the etrasimod 2 mg group compared to placebo (least square mean difference, 0.99; 90% CI, 0.30–1.68; *p* = 0.009) but not in the etrasimod 1 mg group. Compared to placebo, the etrasimod 2 mg group had higher rates of: endoscopic improvement (41.8 vs. 17.8%; *p* = 0.003), improvement in the total Mayo score (*p* = 0.010) and improvement in the 2 component Mayo score (*p* = 0.002) (Sandborn et al., 2020g). Histologic remission (defined as Geboes score <2) was an exploratory endpoint of the trial and was significantly higher in the etrasimod 2 mg group compared to placebo (19.5 vs. 6.1%; *p* = 0.03) (Sandborn et al., 2020g).

In this trial there was significantly higher rates of discontinuation of drug due to AEs in the etrasimod groups (n = 7) compared to placebo (n = 0) (Sandborn et al., 2020g). The most common AEs other than disease worsening are upper respiratory tract infections, nasopharyngitis and anemia. In etrasimod 2 mg group, transient first-degree heart block occurred in two patients and type 1 s degree heart block was noted in one patient. The authors report that all three patients had evidence of atrioventricular block noted on electrocardiography prior to receiving the study drug (Sandborn et al., 2020g).

Phase III induction and maintenance trials of etrasimod for moderate to severe UC are currently recruiting (NCT03996369, NCT03945188, NCT03950232, NCT04176588). A phase II/III study of etrasimod induction and maintenance in CD has recently opened and is currently recruiting (NCT04173273).

#### Amiselimod (MT-1303)

Amiselimod is an oral S1P receptor modulator with higher selectivity for S1PR1 than other S1P receptors. Phase II study of amiselimod demonstrated that is well tolerated and potentially effective for multiple sclerosis (Kappos et al., 2016). A phase II randomized, placebo-controlled trial of amiselimod in CD (NCT02378688) was completed and at this time results are not published. A proof of concept study randomized 78 patients to either amiselimod 0.4 mg (n = 40) or placebo (n = 38) (D’Haens et al., 2019). The primary outcomes of clinical response (decrease in CDAI by 100 points) at week 12 was achieved in 48.7 vs. 54.1% in the placebo group. No significant differences were noted in the fecal calprotectin and CRP in the two groups (D’Haens et al., 2019).

### Phosphodiesterase 4 Inhibitors

Phosphodiesterases (PDE1-PDE11) are a group of intracellular enzymes that catalyze the breakdown of cyclic adenosine monophosphate (cAMP) and cyclic guanosine monophosphate (cGMP). PDE4 catalyzes the breakdown of 3, 5′ cAMP in multiple cell types including T-cells, macrophages and monocytes. This leads to activation of nuclear transcription factor kappaB (NF-κB) which eventually promotes downstream proinflammatory effects. Therefore, inhibition of PDE4 can lead to suppression of NF-κB and subsequently reduce TNF-α mRNA expression and production of nitric oxide and also increase synthesis of anti-inflammatory cytokines such as IL-10 and IL-6 (Ollivier et al., 1996; Gobejishvili et al., 2008; Kwak et al., 2005; Platzer et al., 1999). Therefore, there has been interest in developing PDE4 inhibitors as therapeutic agents in IBD.

#### Apremilast

Apremilast is an oral PDE4 inhibitor that is currently approved by the US FDA for the treatment of psoriasis and psoriatic arthritis. A double-blind, phase II trial randomized UC patients to apremilast 30 mg (n = 57), apremilast 40 mg (n = 55) or placebo (n = 58) twice daily (Danese et al., 2020b). The primary endpoint of clinical remission (defined as total Mayo UC score ≤2; no individual score >1) was achieved in 31.6% in the 30 mg group (*p* = 0.01 compared to placebo), 21.8% in the 40 mg group compared to 12.1% in the placebo group (*p* = 0.27) (Danese et al., 2020b). Endoscopic response (defined as a decrease in the Mayo endoscopic subscore by ≥ 1) was achieved in 41.4% of patients in the placebo group compared to 73.7% in the 30 mg group (*p* < 0.0001) and 47.3% in the 40 mg group (*p* = 0.69). Both the 30 and 40 mg apremilast groups showed greater reduction in CPR and fecal calprotectin compared to placebo. Although the primary endpoint was not reached in this trial, post hoc analysis demonstrated that a significantly higher proportion of patient in both apremilast groups had a Mayo endoscopic subscore of ≤1 compared to placebo (Danese et al., 2020b).

In terms of safety, the most common side effect reported in the apremilast groups were headaches at 25.5% in the 40 mg group and 21.1% in the 30 mg group compared to 6.9% in the placebo group. Serious AE rate was 2.4% in the placebo group and 1.8% in the 40 mg apremilast group (1 patient who experienced pancreatitis but thought not to be related to study drug) (Danese et al., 2020b). Currently, a phase III UC trial of apremilast has not been registered.

### Cobitolimod

Cobitolimod is an oligodeoxynucleotide that is DNA-based and binds to Toll-like receptor 9 (TLR9) on lymphocytes and antigen presenting cells. It activates TLR 9 which lead to induction of regulatory T-cells that produce anti-inflammatory IL-10 in addition to suppressing proinflammatory TH-17 cells. Cobitolimod is a topical agent and is supposed to have low systemic absorption.

A double-blind study (COLLECT) randomized 131 patients with moderate to severe UC to two topically administered doses (during colonoscopy) of cobitolimod 30 mg (n = 87) or placebo (n = 44) (Atreya et al., 2016). The primary endpoint of induction of clinical remission at week 12 (defined as Clinical Activity Index (CAI) ≤4) was 44.4% in the cobitolimod group vs. 46.5% in the placebo group (*p* = 0.91) (Atreya et al., 2016). However, the study did meet multiple secondary endpoints including symptomatic remission, clinical remission with mucosal healing and histologic improvement.

A dose ranging, double-blinded phase IIb study (CONDUCT) randomized 213 patients with moderate to severe left-sided UC into five different treatment arms that received drug or placebo via rectal enemas: cobitolimod 31 mg, 125 mg, or 250 mg at weeks 0 and 3; cobitolimod 125 mg at weeks 0, 1,2 and 3 or placebo (Atreya et al., 2020). The primary endpoint of clinical remission at 6 weeks (defined as Mayo subscore of 0 for rectal bleeding, 0 or 1 for stool frequency + ≥ 1-point decrease from baseline, endoscopy subscore of 0 or 1-excluding friability) was achieved in the cobitolimod 250 mg group at 21% (n = 9) vs. 7% (n = 3) in the placebo group (*p* = 0.025) (Atreya et al., 2020). The rest of the dosing groups did not achieve the primary endpoint compared to placebo. Cobitolimod was well tolerated with an adverse event rate across the dosing groups from 25 to 43% compared to 48% in the placebo group (Atreya et al., 2020). Phase 3 studies are planned for UC (not yet registered at this time).

## Conclusion

In conclusion, the armamentarium of IBD therapeutic agents is ever expanding. This review highlights some of these novel and emerging therapies. For example, there has been significant development in the small molecules class of agents such as JAK inhibitors and S1P receptor modulators. These agents offer the advantages of ease of administration as they are oral agents and do not have the downside of immunogenicity and antibody formation seen with some of the IV/SC biologics. Some of these agents such might also have the possibility of more rapid onset of action as seen with tofacitinib. In addition, some of these agents will likely have the potential of having a more favorable safety profile compared to anti-TNF agents. This includes for example the development of the intestinally restricted pan-JAK inhibitor (TD-1473), anti-integrins/anti-MAdCAM therapies and IL-23 therapies. In the era of increasing therapeutic agents to treat IBD, the challenge will the ability to select the best agent for the individual patient. This calls for the development of precision/personalized medicine approach to the treatment of IBD.

## Disclosures

BA-B is on the Speaker Bureau of Takeda and AbbVie. RS is on the Speaker Bureau of and received clinical research grant support from AbbVie, and is on the advisory board of Pfizer. DP has no conflicts to disclose.

## Abbreviations

AE, adverse event; CD, crohn’s disease; CRP, C-reactive protein; FDA, food and drug administration; IBD, inflammatory bowel disease; IL, interleukin; IFN, interferon; IV, intravenous; JAK, janus kinase; NF-κB, nuclear transcription factor kappaB; PRO, patient reported outcome; S1P, sphingosine 1-phosphate; SC, subcutaneous; TNF-α, tumor necrosis factor alpha; TYK, tyrosine kinase; UC, ulcerative colitis.
